# Functional and phenotypical analysis of IL‐6‐secreting CD4^+^ T cells in human adipose tissue

**DOI:** 10.1002/eji.201747037

**Published:** 2018-01-29

**Authors:** Anja J. de Jong, Sabrina Pollastro, Joanneke C. Kwekkeboom, Stefan N. Andersen, Annemarie L. Dorjée, Aleida M. Bakker, Fawaz Alzaid, Antoine Soprani, Rob G.H.H. Nelissen, Jan B. Mullers, Nicolas Venteclef, Niek de Vries, Margreet Kloppenburg, René E.M. Toes, Andreea Ioan‐Facsinay

**Affiliations:** ^1^ Department of Rheumatology Leiden University Medical Centre, Leiden The Netherlands; ^2^ Department of Experimental immunology Academic Medical Center Amsterdam The Netherlands; ^3^ Department of Clinical Immunology & Rheumatology ARC | Academic Medical Center Amsterdam The Netherlands; ^4^ Institut National de la Santé et de la Recherche Médicale (INSERM) UMRS 1138 Sorbonne Universités Paris France; ^5^ Clinique Geoffroy Saint‐Hilaire Ramsey General de Santé Paris France; ^6^ Department of Orthopaedics Leiden University Medical Center Leiden the Netherlands; ^7^ Department of Orthopaedic Surgery Alrijne Hospital Leiden the Netherlands; ^8^ Sorbonne Paris Cité Université Paris Descartes Université Paris Diderot Paris France; ^9^ Centre de Recherche des Cordeliers Paris France

**Keywords:** Adipose tissue, CD4, IL‐6, Osteoarthritis, T cells

## Abstract

Emerging evidence indicates that a dynamic interplay between the immune system and adipocytes contributes to the disturbed homeostasis in adipose tissue of obese subjects. Recently, we observed IL‐6‐secretion by CD4^+^ T cells from the stromal vascular fraction (SVF) of the infrapatellar fat pad (IFP) of knee osteoarthritis patients directly ex vivo. Here we show that human IL‐6^+^CD4^+^ T cells from SVF display a more activated phenotype than the IL‐6^−^ T cells, as evidenced by the expression of the activation marker CD69. Analysis of cytokines secretion, as well as expression of chemokine receptors and transcription factors associated with different Th subsets (Treg, Th1, Th2, Th17 and Tfh) revealed that IL‐6‐secreting CD4^+^ T cells cannot be assigned to a conventional Th subset. TCRβ gene analysis revealed that IL‐6^+^ and IL‐6^−^CD4^+^ T cells appear clonally unrelated to each other, suggesting a different specificity of these cells. In line with these observations, adipocytes are capable of enhancing IL‐6 production by CD4^+^ T cells. Thus, IL‐6^+^CD4^+^ T cells are TCRαβ T cells expressing an activated phenotype potentially resulting from an interplay with adipocytes that could be involved in the inflammatory processes in the OA joint.

## Introduction

Obesity is an increasing problem in the Western world, and is associated with several diseases. These include the long‐known metabolic and cardiovascular disorders 1, 2, but also the more recently described associations with inflammatory diseases, such as rheumatoid arthritis, osteoarthritis and inflammatory bowel diseases 3, 4, 5. Although for most of the associations, the underlying mechanisms are unclear, adipose tissue inflammation is believed to play an important role in obesity‐related disorders.

The expansion of adipose tissue during weight gain and development of obesity is accompanied by a switch from an anti‐inflammatory state to a pro‐inflammatory state of the adipose tissue 1. The precise sequence of events leading to this switch is incompletely understood, but several studies suggest that changes in both adipocytes and immune cells are involved in this process. Obesity is accompanied by an increase in adipocyte number, size and death 6, 7, 8 resulting in hypoxia and infiltration of immune cells, including T cells 9, 10. Mouse studies revealed an accumulation of Th1 and CD8^+^ T cells in the obese adipose tissue, at the expense of Treg and Th2 cells 9, 11, 12, 13. Moreover, depletion studies indicated that Th2 and Treg are involved in maintaining insulin sensitivity, while Th1 and CD8 T cells contribute to insulin resistance and adipose tissue inflammation 9, 11, 12.

Although limited knowledge is available about adipose tissue in humans, both CD4^+^ and CD8^+^ T cells were shown to be present more abundantly in obesity 13, 14, 15. The observations regarding Th subsets and their association with obesity are, however, less clear in humans. Similar to observations in mice, Th1 cells have been shown to be enhanced in obesity 11, 13 and to outnumber Treg cells 11 in some studies, while in other studies Th17 cells were enhanced with no enhancement of Th1 or Th2 in adipose tissue 15, 16. Moreover, the percentage of Th1 cells correlated positively with insulin resistance 13 and the percentage of Th2 cells correlated inversely with insulin resistance 17, 18 in some studies. Overall, studies in humans support a possible role for pro‐inflammatory Th cells, such as Th1 cells in adipose tissue inflammation and subsequent insulin resistance. Although the mechanisms underlying the accumulation of certain Th subsets in adipose tissue and their downstream effects are still unclear, some murine studies showed that CD4^+^ T cells in adipose tissue have a limited TCR‐repertoire 9, 11, 12, 14, suggesting that they underwent clonal expansion. Whether these cells have expanded in the adipose tissue and which antigen these cells recognize is unknown. Moreover, it is unknown what the phenotype of these cells is and which cytokines they secrete. Recently, we demonstrated that CD4^+^ T cells from the infrapatellar fat pad (IFP), an adipose tissue located in the knee, secrete IL‐6 directly ex vivo 19. These findings were unexpected considering that there was no additional stimulus, indicating recent activation of the T cells. This could suggest that these cells recognize adipose tissue antigens and could play a role in adipose tissue inflammation. Therefore, the aim of this study was to further characterize the IL‐6^+^CD4^+^ T cells of IFP, to gain a better understanding of the possible role of these cells in adipose tissue.

## Results

### CD4^+^ T cells from IFP secrete IL‐6 ex vivo

**Figure 1:**
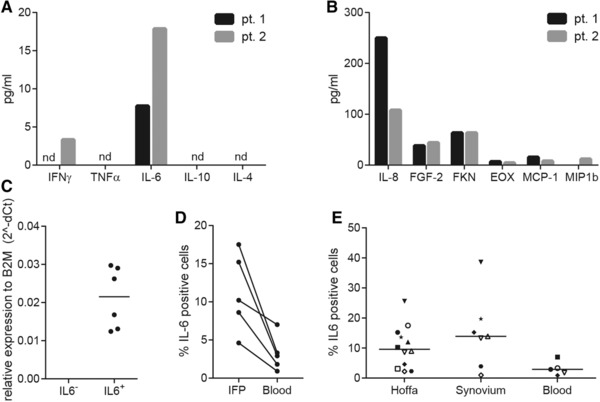
CD4^+^ T cells from IFP secrete IL‐6 ex vivo. Spontaneous cytokine production by CD4^+^ T cells from SVF was confirmed and expanded by testing supernatants of sorted CD4^+^ T cells (pt 1: 12878, pt 2: 32347 cells/well) with luminex for 42 cytokines (A and B) (*N* = 2). Data are representative of 2 independent experiments. IL‐6^+^ and IL‐6^−^CD4^+^ T cells were isolated from SVF using an in‐house generated capture complex (Supporting Information Fig. 1), cDNA was generated and IL‐6 mRNA levels were determined (*N* = 1)(C). Median is shown. Data are from 6 groups of 20 cells from 1 independent experiment (performed in triplo). The presence of IL‐6^+^CD4^+^ T cells was also determined in synovium and blood by flow cytometry (see gating strategy Supporting Information Fig. 2) (*N* = 5–12). Paired IFP and blood samples are depicted in D, a summary of all obtained tissues is depicted in E. Each symbol represents a patient. Median is shown. Data are representative of 5–12 independent experiments with 1 patient per experiment.

Secretion of IL‐6 by CD4^+^ T cells present in the SVF was confirmed by multiplex ELISA using culture supernatant of sorted CD4^+^ T cells from IFP (Fig. 1A). Next to IL‐6, secretion of IFNγ, IL8, FGF‐2, fractalkine, eotaxin, MCP‐1 and MIP1β was also evident in isolated CD4^+^ T cells (Fig. 1B). To determine whether the previously found IL‐6^+^CD4^+^ T cells population was the source of the IL‐6 in the supernatant, we isolated IL‐6^+^CD4^+^ T cells from SVF using an in‐house developed capture complex (Supporting Information Fig. 1A). The specificity of the isolation procedure was validated by ELISA and the results indicated that IL‐6 could only be detected in the culture supernatant of IL‐6^+^CD4^+^ T cell population and that IL‐6^+^CD4^+^ T cells produce 0.0005 – 0.003 pg of IL‐6 per cell (Supporting Information Fig. 1B). However, the sensitivity of this procedure was lower than intracellular cytokine staining (data not shown). Moreover, q‐PCR analyses indicated that mRNA expression of IL‐6 could only be detected in the IL‐6^+^CD4^+^ T cells and not in IL‐6^−^CD4^+^ T cells (Fig. 1C).

Intriguingly, a similar population was present in synovial tissue and IFP in paired samples, and only to a lower extend in peripheral blood, as analysed when available (Fig. 1D and E). This population could also be detected in subcutaneous adipose tissue (SCAT) and visceral adipose tissue (VAT) of patients undergoing bariatric surgery (Supporting Information Fig. 3). Thus, our data indicate that the population of CD4^+^ T cells capable of producing IL‐6 without additional stimulus ex vivo is not restricted to the IFP.

### Phenotypic characterization of IL‐6^+^CD4^+^ T cells

**Figure 2:**
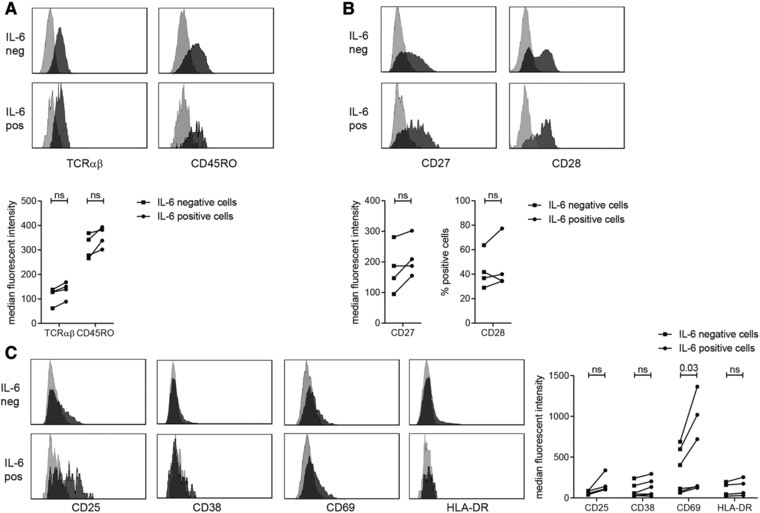
Phenotypic characterization of IL‐6^+^CD4^+^ T cells. Unstimulated IL‐6^+^CD4^+^ T cells and IL‐6^−^CD4^+^ T cells from SVF were characterized by flow cytometry (see gating strategy Supporting Information Fig. 2) for general T cell markers (A) (*N* = 4), co‐stimulatory markers (B) (*N* = 4) and activation markers (C) (*N* = 4–6). Data are examples of stainings and summary graphs of all patients tested in 4–6 independent experiments with 1 patient per experiment: light grey is isotype, dark grey is staining, Wilcoxon's singed rank test was used to compare differences between groups. ns: non‐significant.

To obtain insight into the possible function of this enigmatic T‐cell population, we performed an extensive phenotypic characterization. IL‐6^+^CD4^+^ T cells expressed TCRαβ and CD45RO (Fig. 2A) indicating that they are conventional αβ memory T cells. Furthermore, IL‐6^+^CD4^+^ T cells expressed both CD27 and CD28 (Fig. 2B). Since IL‐6^+^CD4^+^ T cells produced cytokines without additional stimulation, we hypothesized that these cells could be recently activated. Therefore, we assessed the activation states of these cells and found that IL‐6^+^CD4^+^ T cells expressed CD25 and CD69, and little CD38 and HLA‐DR (Fig. 2C). Moreover, expression of CD69 appeared to be higher on IL‐6^+^CD4^+^ T cells than their IL‐6^−^CD4^+^ counterparts. The addition of IL‐2 did not affect IL‐6, CD25 and CD69 expression (Supporting Information Fig. 4). Similarly, adipose tissue secreted factors were not able to enhance CD69 expression by CD4^+^ T cells, as assessed by addition of fat‐conditioned medium (FCM) to peripheral blood mononuclear cells for 24 and 48 h (Supporting Information Fig. 5), suggesting that the expression of CD69 is not induced by adipose tissue secreted factors. Besides being an activation marker, CD69 is also expressed on tissue resident T cells 20, 21. However, neither IL‐6^+^ nor IL‐6^−^CD4^+^ T cells expressed Hobit (data not shown), a transcription factor associated with tissue resident T cells 22. In conclusion, IL‐6^+^ T cells from IFP are a population of memory CD4^+^ T cells, present in IFP in an activated state.

### IL‐6^+^CD4^+^ T cells cannot be categorized as a conventional T helper subset

**Figure 3:**
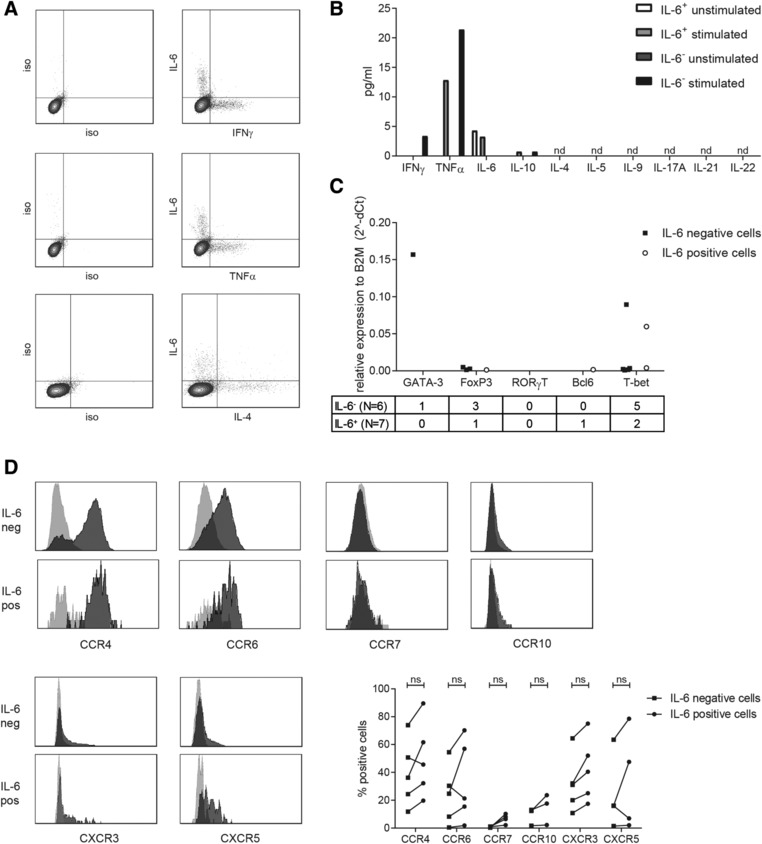
IL‐6^+^CD4^+^ T cells cannot be categorized as a conventional T helper subset. SVF cells were stimulated overnight with PMA/ionomycin and simultaneous cytokine production of CD4^+^ T cells was assessed by flow cytometry (A) (*N* = 12). Data are representative of 12 independent experiments. Unstimulated and αCD3/αCD28‐induced cytokine production by isolated IL‐6^+^CD4^+^ T cells and IL‐6^−^CD4^+^ T cells (8700 cells per well) from SVF was determined (B) (*N* = 1). Data are representative of 1 experiment (performed in duplo). Unstimulated IL‐6^+^CD4^+^ T cells and IL‐6^−^CD4^+^ T cells from SVF were isolated and the expression levels of transcripts was determined by qPCR (C)(*N* = 1) Data are from 9 to 10 groups of 20 cells from 1 independent experiment (performed in triplo). Numbers under the graph represent the number of groups of 20 cells positive for the transcription factor. Chemokine receptor expression was determined on unstimulated IL‐6^+^CD4^+^ T cells and IL‐6^−^CD4^+^ T cells from SVF by flow cytometry(D) (*N* = 3–5) Data are examples of all stainings and summary graphs of all patients tested in 3–5 independent experiments performed: light grey is isotype, dark gray is staining, Wilcoxon's singed rank test was used to compare differences between groups.

Next, we investigated whether IL‐6^+^CD4^+^ T cells also expressed other cytokines that are classically assigned to certain helper subsets. Intracellular cytokine staining revealed that most IL‐6 producing T cells do not secrete other cytokines such as IFNγ, TNF‐α, and IL4 (Fig. 3A). These data were expanded by milliplex analysis, which indicated that IL‐6^+^CD4^+^ T cells were able to secrete TNF‐α and negligible amounts of IL‐10, but no IFNγ, IL‐4 IL‐5, IL‐9, IL‐10, IL‐17A, IL‐21 or IL‐22 upon stimulation with αCD3/αCD28 (Fig. 3B). Furthermore, analysis of transcription factors T‐bet, GATA‐3, RORγT, FoxP3 and Bcl6 revealed that most IL‐6^+^ T cells (4 out of 7 groups of 20 cells) did not express any of the tested transcription factors, while all groups of IL‐6^−^ T cells expressed at least one transcription factor (Fig. 3C). One group of IL‐6^+^ T cells expressed FoxP3, one expressed T‐bet and one expressed both FoxP3 and Bcl6. These data indicate that the majority of the IL‐6^+^CD4^+^ T cells could not be assigned to any conventional T helper subset. Likewise, chemokine receptor expression analyses showed that IL‐6 producing T cells expressed a variety of chemokine receptors (Fig. 3D), precluding their unambiguous assignment to a certain T helper subset defined by their chemokine receptor expression. Together, these data suggest that the IL‐6^+^CD4^+^ T cells are not a conventional T helper subset.

### TCRβ repertoire of IL‐6^+^CD4^+^ T cells and IL‐6^−^CD4^+^ T cells

**Figure 4:**
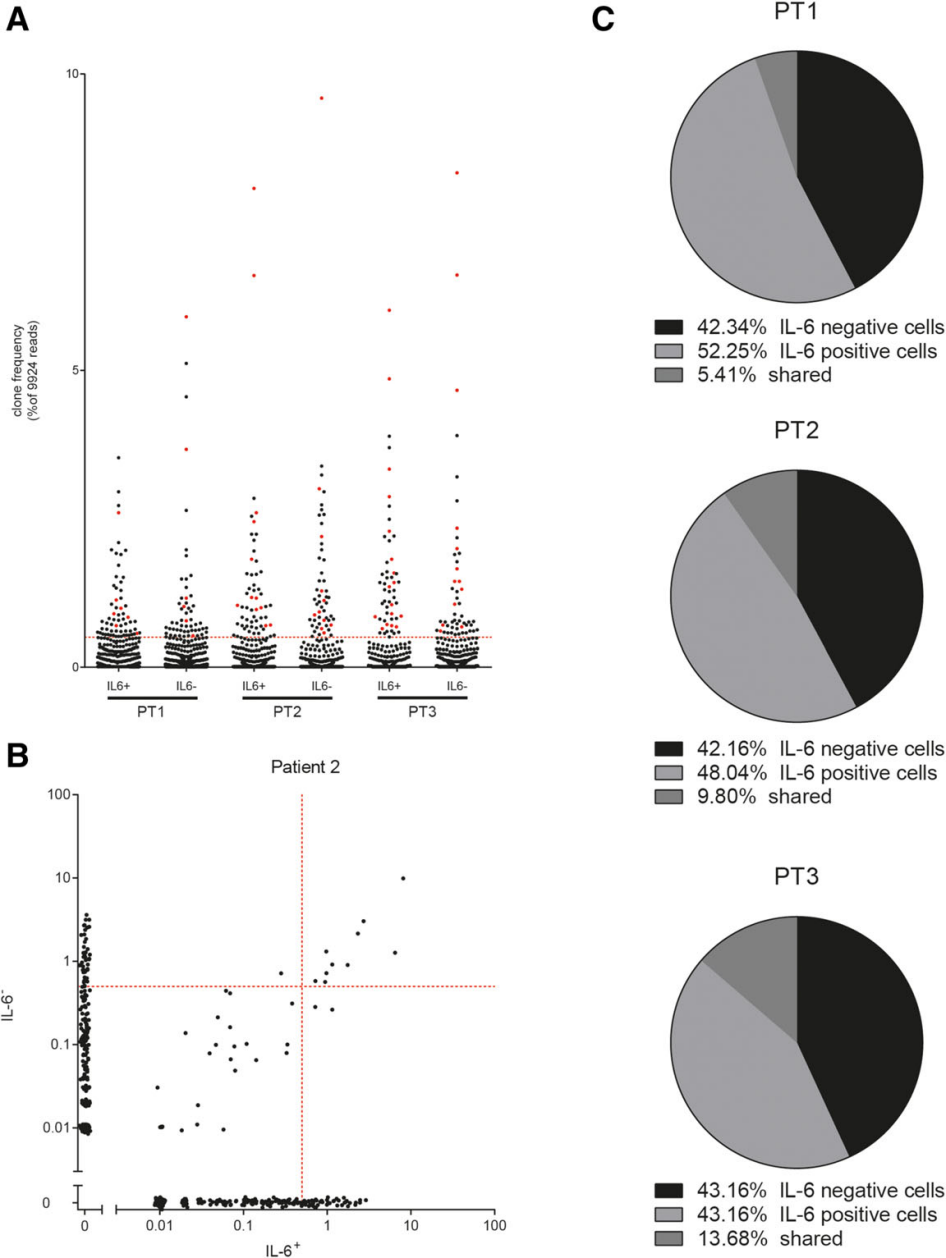
TCRβ repertoire of IL‐6^+^CD4^+^ T cells and IL‐6^−^CD4^+^ T cells. Spontaneous IL‐6^+^CD4^+^ T cells and IL‐6^−^CD4^+^ T cells were isolated from SVF and the abundance and distribution of the TCRβ repertoire was determined (*N* = 3). Scatterplot representing the clonal repertoire of the IL‐6^+^CD4^+^ T cells and IL‐6^−^CD4^+^ T cells (A). Each dot represents a single clone defined by its V‐J‐CDR3 combination (V = variable gene; J = joining gene; CDR3 = complementary determining region 3), red dots represent clones shared between the IL‐6^+^CD4^+^ T cells and IL‐6^−^CD4^+^ T cells populations within each patient and the gray horizontal dashed line represents the frequency limit of 0.5% for the definition of highly expanded clones (HECs). (B). Scatter plots of overlapping clones between the IL‐6^+^ (X‐axis) and the IL‐6^−^ (Y‐axis) CD4^+^ T cells population for one of the three patients analysed. Each dot represents a single clone. Dots along the axes represent unshared clones. Grey dotted lines indicate the frequency limit of 0.5% for the definition of highly expanded clones (HECs). Pie charts showing the percentage of the HECs present only in the IL‐6^+^CD4^+^ T cells population (light gray), only in the IL‐6^−^CD4^+^ T cells population (black), and shared between both populations (dark grey) (C). Data depict 3 independent experiments out of 3 performed with 1 patient sample per experiment.

The fact that IL‐6^+^CD4^+^ T cells from SVF display an activated state suggests that they have recently encountered antigen. Therefore, we determined the abundance and distribution of TCRβ rearrangements in the IL‐6^+^ and the IL‐6^−^CD4^+^ T cell populations. The overall TCRβ repertoire did not show major differences between IL‐6^+^ and IL‐6^−^CD4^+^ T cells populations (Fig. 4A). Figure 4B shows a representative example of the clonal overlap between the IL‐6^+^ and the IL‐6^−^CD4^+^ T cell populations from the same patient, while a summary of all 3 patients is presented in Fig. 4C. In the pool of highly expanded clones (HECs), i.e. clones with read frequency above 0.5%, only a low number of TCRβ rearrangements were shared between the IL‐6^+^ and IL‐6^−^CD4^+^ T cell populations in all 3 patients (Fig. 4A, red dots and Fig. 4C). The percentage of HECs shared between the IL‐6^+^ and IL‐6^−^CD4^+^ T cell populations varied per patient and was between 5 and 15% (Fig. 4C) indicating that the IL‐6^+^ and IL‐6^−^ populations have a different TCRβ usage, thus are clonally unrelated.

### Adipocytes enhance IL‐6 production in CD4^+^ T cells

**Figure 5:**
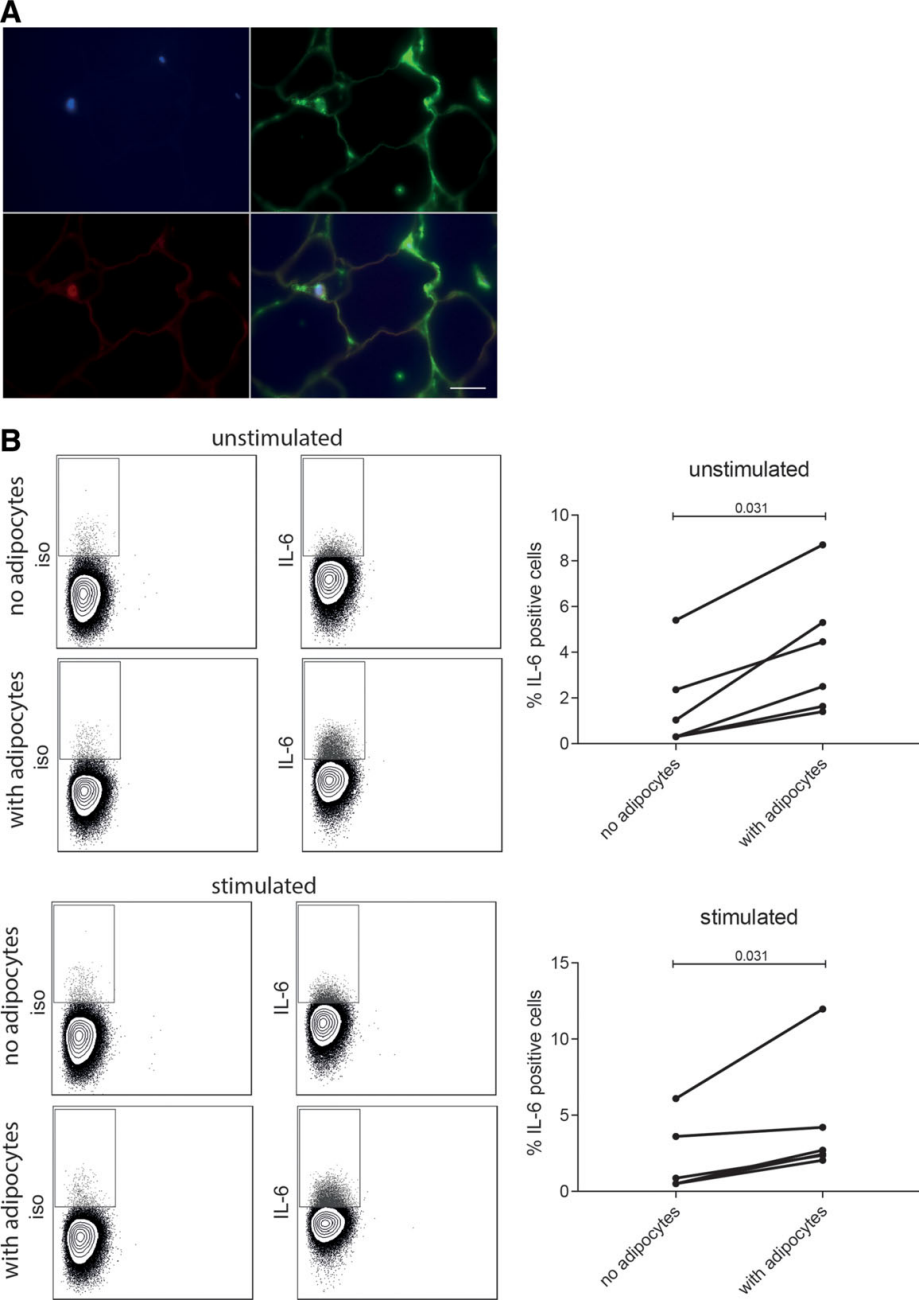
IL‐6^+^CD4^+^ T cells are in close proximity with adipocytes and adipocytes can enhance IL‐6 secretion in CD4^+^ T cells. IFP was stained for CD3 (in red) and IL‐6 (in green) (A)(*N* = 2). Scale bar: 10 μm. One representative image is shown of 2 independent experiments performed with 1 slide per donor. Adipocytes and CD4^+^ T cells were co‐cultured overnight after which intracellular staining for IL‐6 was performed (B)(*N* = 6). CD4^+^ T cells were either unstimulated or αCD3/αCD28 stimulated. Examples of the staining is shown and a summary of all donors tested, each line represents a T‐cell‐adipocyte combination. Wilcoxon's signed rank test was used to compare differences between groups. Data are pooled from 6 independent experiments with 1–2 donors per experiment (performed in triplo).

Immunofluorescence staining of IFP indicated that IL‐6^+^CD4^+^ T cells were usually scattered through adipocytes and did not form clusters with other immune cells within the tissue (Fig. 5A). Therefore, we hypothesized that adipocytes might stimulate IL‐6 production by T cells. To address this hypothesis, we co‐cultured adipocytes together with peripheral blood CD4^+^ T cells overnight and assessed the percentage of IL‐6 T positive cells. Co‐culture of CD4^+^ T cells with adipocytes led to a higher percentage of IL‐6 positive cells compared to CD4^+^ T cells cultured without adipocytes (Fig. 5B). This effect was independent of αCD3/αCD28 stimulation. These data indicate that IL‐6^+^ production by CD4^+^ T cells can be enhanced by adipocytes.

## Discussion

In this study, we investigated IL‐6^+^CD4^+^ T cells present in the SVF of the IFP in knee osteoarthritis patients. Phenotypic characterization indicated that they are conventional (TCRαβ) memory CD4^+^ T cells with an activated phenotype. Their TCRβ repertoire is distinct from IL‐6^−^CD4^+^ T cells and they do not simultaneously secrete other cytokines associated with conventional Th subsets. Furthermore, these IL‐6^+^CD4^+^ T cells are scattered through the adipose tissue and are in close proximity to adipocytes which are capable of enhancing production of IL‐6 in CD4^+^ T cells.

In order to determine whether these cells differ from conventional T helper subsets 23, we determined cytokine production, transcription factor expression and chemokine receptor expression on IL‐6‐producing CD4^+^ T cells. Both IL‐6^+^ and IL‐6^−^CD4^+^ T cells expressed a variety of chemokine receptors, however, no specific combinations were observed that could be used to assign IL‐6^+^CD4^+^ T cells to a specific T helper subset (*data not shown*). Although the variety of chemokine expression could indicate that these IL‐6^+^CD4^+^ T cells are a mixture of T helper subsets, analyses of transcription factor expression makes this hypothesis unlikely, since most of IL‐6^+^CD4^+^ T cells do not express any of the transcription factors associated with conventional Th subsets. Despite the fact that the latter results are based on cells from one patient, they support the finding that these IL‐6^+^CD4^+^ T do not belong to a conventional T helper subset.

TCRβ repertoire analysis revealed that IL‐6^+^CD4^+^ T cells from SVF from IFP have a distinct TCRβ usages compared to their IL‐6^−^CD4^+^ T cells counterparts. This suggests that, despite the fact that both IL‐6^+^ and IL‐6^−^CD4^+^ T cells underwent clonal expansion, the epitopes/antigens they recognize are different. Moreover, the differential cytokine secretion could indicate that IL‐6^+^CD4^+^ T cells were primed under different conditions than their IL‐6^−^ counterparts. Secretion of IL‐6 ex vivo indicates that these cells have been recently activated. Whether these cells are activated in the adipose tissue or elsewhere and then attracted to adipose tissue is unknown. Both mechanisms have been previously described: CCL20 secreted by adipocytes can attract CD4^+^ T cells 24, while the restricted repertoire of T cells in adipose tissue suggested their local expansion 11, 12. Indeed, the number of CCR6^+^ T cells in IFP is highly variable between donors and this might be due to secretion of various levels of CCL20 by IFP adipocytes.

As these IL‐6^+^CD4^+^ T cells express CD69, they could be tissue resident T cells 20, 21. Although less is known about the phenotype of tissue resident CD4^+^ T cells compared to their CD8^+^ counterparts, it has been recently described that Hobit is a transcription factor upregulated in CD8^+^ tissue resident T cells 22. In our study, neither IL‐6^+^CD4^+^ T cells nor their IL‐6^−^ counterparts expressed this marker (*data not shown*). Alternatively, it is possible that CD69 indicates the recent activation of these cells.

Adipocytes have been shown to express MHCII 25, therefore it is conceivable that adipocytes initiate or sustain IL‐6 production by CD4^+^ T cells. Indeed, our data indicate that IL‐6^+^ T cells are in the proximity of adipocytes and adipocytes are capable of enhancing IL‐6 secretion by CD4^+^ T cells. Which mechanisms are employed by adipocytes to enhance the IL‐6 secretion of CD4^+^ T cells remains unknown. Moreover, the possibility that adipocytes enhance the proliferation or survival of IL‐6 producing T cells needs to be addressed in future studies.

Although the function and clinical relevance of these IL‐6 producing T cells in adipose tissue is still unclear, it has been previously shown that IL‐6 can affect adipocytes and enhance lipolysis 26, 27. Our data suggest that T cells are situated in the vicinity of adipocytes and IL‐6 production of T cells can be modulated by adipocytes. Therefore, it is conceivable that IL‐6^+^ T cells could in turn modulate adipocyte function. This is in line with previously published data indicating that a cross‐talk between adipocytes and immune cells in adipose tissue exists 24. It is therefore conceivable that the IL‐6 or other factors secreted by IL‐6^+^ T cells could act as a feed‐back mechanism on adipocytes by limiting their expansion through enhanced lipolysis. This remains, however, to be investigated. Furthermore, the IL‐6 secreted by T cells on a per cell basis is comparable to IL‐6 secreted by OA synoviocytes, indicating that they could contribute to the inflammatory processes in the OA joint.

In conclusion, we have found a population of CD4^+^ T cells that secrete IL‐6 directly ex vivo and are in an activated state. Phenotypic characterization of these cells suggested that they might recognize adipose tissue antigens and could affect adipose tissue function through interaction with adipocytes.

## Materials and methods

### Human subjects

Patients with primary osteoarthritis undergoing knee joint replacement were included into the study (N = 67: 62.7% women, median age 71 years, median (IQR) BMI 29.7 kg/m^2^ (26.7‐33.1). Preoperative blood, IFP, subcutaneous adipose tissue (SCAT) and synovium removed during surgery were obtained after informed consent. Due to limited numbers of cells not all experiments could be performed on each tissue sample. The study was approved by the local ethical committee. Visceral adipose tissue (VAT) and SCAT were obtained from patients undergoing bariatric surgery (N = 3: all male, mean age 48.5 years, mean BMI (SD) 43 kg/m^2^ (1.6). The study was conducted in accordance with the Helsinski Declaration. The Ethics Committee of CPP Ile‐de‐France approved the clinical investigations for all individuals, and written informed consent was obtained from all individuals. Buffy coats were from healthy donors and the study was approved by the local medical ethical committee.

### Cell isolation

Stromal vascular fraction (SVF) was isolated as previously described 28 and was used for flow cytometric analysis or the isolation of CD4^+^ T cells, IL‐6^+^ and IL‐6^−^CD4^+^ T cells. To isolate CD4^+^ T cells from SVF of two patients, cells were stained with Dead cell discriminator kit (Miltenyi Biotec, Bergisch Gladbach, Germany) and with AF‐700‐conjugated CD3, Pacific Blue‐conjugated CD4, APC‐conjugated CD8, PE‐Cy‐7‐conjugated CD14 (all from BD Biosciences, Breda, The Netherlands). Using a FACS Aria CD3^+^CD4^+^CD14^−^CD8^−^ cells were sorted. Cells were plated in a 96‐well plate in DMEM 4.5 g/l glucose/F12/0.5% BSA/15 mM Hepes/glutamax/pen/strep and supernatant was harvested after 1 day of culture. Adipocytes were isolated as previously described 28 and used for co‐cultures with CD4^+^ T cells. PBMCs were isolated from buffy coats of healthy donors by standard ficoll plaque gradient. PBMCs were used for treatment with FCM or for isolation of CD4^+^ T cells. CD4^+^ T cells were purified using magnetic beads labelled with anti‐CD4 (Invitrogen Dynal, Oslo, Norway), followed by removal of the magnetic beads, according to the manufacturer's instructions. The purity of the isolated CD4^+^ T cells was typically above 95%. CD4^+^ T cells were used for co‐cultures with adipocytes.

### Flow cytometric analysis

SVF was plated overnight in a 6‐wells plate at a density of 5 × 10^6^ cells/well maximum in DMEM 4.5 g/l glucose/F12/0.5% BSA/15 mM Hepes/glutamax/pen/strep (Invitrogen) (medium) supplemented with 50 IU/mL IL‐2 (Peprotech). For polyclonal activation of T cells 20 ng/mL phorbol myristate acetate (PMA) and 200 ng/mL ionomycin was added after 16 h and incubated for 5 h. Brefeldin A 10 μg/mL was added after 1 h, where after cells were harvested and surface and intracellular stainings were performed. For ex vivo determination of cytokine production cells were incubated overnight in medium supplemented with 3 μg/mL Brefeldin A and 50 IU/mL IL‐2. Cells were harvested and surface and intracellular stainings were performed. Exclusion of dead cells was performed in experiments when possible using the dead cell discrimination kit (Miltenyi Biotec), according to the manufacturer's specifications. Cells were first stained with antibody mixes containing the following surface markers: AF‐700‐conjugated CD3, Pacific blue‐conjugated CD4, FITC‐conjugated CD8, HLA‐DR, TCRαβ, APC‐conjugated CD8, CD28, CD45RO, CCR10, CXCR3, AF‐647‐conjugated CXCR5, PE‐Cy‐5‐conjugated CD69, PE‐Cy‐7‐conjugated CD14, CD25, CD27, CCR4, PercpCy5.5‐conjugated CD38, CCR6, CCR7 and PE‐Texas red‐conjugated CD69 (all from BD Biosciences). Next, intracellular cytokines were detected using Cytofix/Cytoperm Fixation/Permeabilization Solution Kit (BD Biosciences) according to manufacturer's instructions. Antibody mixes for intracellular stainings contained the following cytokine antibodies: PE‐conjugated Abs to interferon γ (IFNγ), IL10, tumour necrosis factor α (TNFα), IL‐6, Pe‐Cy‐7‐conjugated IL4, FITC‐conjugated IL‐6 and the appropriate isotype controls (all BD Biosciences except the Ab to PE‐conjugated IL‐6 which was from eBioscience). Cells were fixed with paraformaldehyde and analysed with LSRII flow cytometer using Diva 6 software (BD Biosciences).

### Isolation of IL‐6^+^ and IL‐6^−^CD4^+^ T cells from SVF

To isolate IL‐6^+^ cells an IL‐6 capture complex was generated. The IL‐6 capture complex consists of biotin labelled CD45 and biotin labelled IL‐6 both complexed to avidin. First 1 μL of 200 μg/mL biotin labelled CD45 and 10 μL of 500 μg/mL biotin labelled IL‐6 were combined and vortexed. Next, 1 μL of 5 mg/mL avidin was added and mixed again. The capture complex was incubated for 10 min at room temperature and vortexed before use. A total of 6 μL of the capture complex was added to 30 μL PBS/2%FCS containing 1 × 10^6^ isolated SVF cells and incubated for 15 min. SVF was then incubated with complete medium with 50 IU/mL IL2 (Peprotech) overnight at 37°C under continuous rolling. Next day SVF was washed with PBS/2% FCS, where after dead cells were stained with Dead cell discriminator kit (Miltenyi Biotec, Bergisch Gladbach, Germany) and with AF‐700‐conjugated CD3, Pacific Blue‐conjugated CD4, APC‐conjugated CD8, PE‐Cy‐7‐conjugated CD14 (all from BD Biosciences, Breda, The Netherlands) and PE‐conjugated IL‐6 (eBiosciences, Vienna, Austria). Using a FACS Aria both CD3^+^CD4^+^CD14^−^CD8^−^IL‐6^+^ and CD3^+^CD4^+^CD14^−^CD8^−^IL‐6^−^ were sorted. From one patient IL‐6^+^ and IL‐6^−^ sorted cells were plated in a 96‐well plate in complete medium either unstimulated or stimulated with 5 μg/mL plate‐bound (pb.) αCD3 (clone OKT3, eBioscience, San Diego, USA) and 1 μg/mL soluble (sol.) αCD28 (Sanquin, Amsterdam, The Netherlands). Supernatant was harvested after 3 days of culture.

### Preparation of cDNA for real‐time quantitative polymerase chain reaction (qPCR)

Sorted cells were processed using the Smart‐seq2 protocol 29 with minor changes. A total of 20 sorted cells were used per reaction. Reverse transcription was carried out with SMartscribe Reverse transcriptase (100 U/mL), without adding MgCl. ERCC RNA Spike‐In Mix (ThermoFisher) controls were used to control for variability between samples. Furthermore, pre‐amplification was carried out by denaturing at 95°C and using 19 PCR cycles. Purification of the resulting cDNA was performed with Ampure XP beads using a beads to sample ratio of 0.8:1. Real‐time qPCR was performed with primers specific for IL‐6, FoxP3, RORγt, GATA‐3 B2M and LMNA as well as ERCC‐0074 and ERCC‐0096 which were primers specific for the ERCC RNA Spike‐In Mix to control for variability between samples. The cDNA was 1:10 diluted (1:100 for B2M and LMNA) and qPCR was performed using SensiFast Sybr (Bio‐line) and primers at a concentration of 250 nM or 375 nM for GATA‐3 in a total volume of 8 μL. The qPCR was performed on a real time PCR system (Bio‐Rad CFX‐384) with an activation step of 2 min on 95°C (hot start polymerase activation), a melting temperature of 95°C for 5 s and an annealing temperature of 58°C (ERCC‐0074 and ERCC‐0096), 58°C (B2M and LMNA) or 64°C (IL‐6, FoxP3, RORγt, GATA‐3) for 10 s and an elongation step for 25 s on 72°C for 40 cycles. Melting curves were performed from 65 to 95°C to test specific binding of SensiFast Sybr at the end of the protocol. For T‐bet and Bcl6 cDNA was 1:10 diluted and qPCR was performed using primers at a concentration of 200 nM and SensiFast Sybr in a total volume of 10 μL. Again, a real time PCR system was used to perform the qPCR with an activation step of 3 min on 95°C, 40 cycles of melting temperature of 95°C for 3 s, an annealing temperature of 60°C for 30 s and an elongation step for 10 s on 95°C . At the end of the protocol melting curves were performed from 65°C to 95°C to test specific binding of SensiFast Sybr.

### High‐throughput sequencing

RNA extraction, cDNA synthesis and linear amplification were performed as previously described 30, 31. Briefly, TCRβ repertoire was amplified in two steps. First, using a mix of primers covering all the functional TCRβ variable genes a linear amplification was performed. The amplification product was used, after purification, in a normal PCR to obtain amplicons spacing from the TCRβ variable region to the TCRβ constant region. Amplicons were purified, quantified, prepared for sequencing according to the sequencing platform manufacturer's manual and sequenced on a Roche Genome Sequencer FLX (titanium platform).

### CDR3 sequence analysis

The bioinformatic pipeline used to extract TCRβ sequences was described previously 30, 31. In short, TCRβ reads obtained from the sequencing platform are “fingerprinted” based on the V‐J‐CDR3 identified in the sequence (V = Variable gene, J = Joining gene, CDR3 = Complementary Determining Region 3). TCRβ sequences with unique fingerprint are regarded as clones. The frequency of each clone is calculated based on the total amount of reads. Clones with frequencies above 0.5% of the total repertoire are considered as Highly Expanded Clones (HECs).

### IL‐6^+^ T cell tissue staining

IFP pieces were fixed in 4% formalin overnight followed by storage in EtOH, before embedding in paraffin. Four micrometre sections were deparaffinised and rehydrated. Antigen retrieval was performed with EDTA (pH 9) (DAKO, USA) at 96°C for 30 min. After cooling, sections where blocked with blocking solution (10% Normal Donkey Serum (DS) in 1% BSA/PBS) for 30 min at RT. Sections were incubated overnight at 4°C with monoclonal mouse anti‐CD3 (DAKO, Glostrub, Denmark) and polyclonal rabbit anti‐IL‐6 (Abcam, Cambridge, UK) in 1% DS/1% BSA/PBS. Sections were then washed with PBS and incubated with polyclonal donkey anti‐mouse IgG Alexa Fluor 568 for CD3 and polyclonal donkey anti‐rabbit IgG Alexa Fluor 488 for IL‐6 for 1 hr at RT. Sections were washed with PBS, dried and covered with Vectashield Hard Set mounting medium with DAPI (Vector laboratories, Burlingame, USA) and analysed on a fluorescence imaging microscope.

### Co‐culture of CD4^+^ T cells with adipocytes of fat conditioned medium

PBMCs were cultured overnight in DMEM 4.5 g/l glucose/F12/0.5% BSA/15 mM Hepes/glutamax/pen/strep (Invitrogen) (medium) supplemented with 50 IU/mL IL‐2 (Peprotech). Next day suspension cells were harvested and cultured in a density of 500,000 cells/well in 24‐well plates in 1.2 mL medium. Fat conditioned medium (FCM) was generated as previously described 28 and added to the cells in final ratio of 1:2. Stimulation with 0.5 μg/mL with Phytohaemagglutinin (PHA) was used as positive control. After 24 and 48 h of incubation cells were harvested and expression of CD69 was determined on CD3^+^CD4^+^ cells, upon staining with Dead cell discriminator kit and FITC‐conjugated CD3, APC‐conjugated CD4 and PE‐Texas red‐conjugated‐conjugated CD69. Isolated CD4^+^ T cells were cultured overnight at a density of 200 000 cells/well in 96‐well plates in 200 μL medium. CD4^+^ T cells were co‐cultured with 20 μL of isolated adipocytes. Cultures were performed in absence or presence of 5 μg/mL plate‐bound anti‐CD3 antibody (clone OKT3, eBioscience, San Diego, USA) and 1 μg/mL soluble anti‐CD28 antibody (Sanquin, Amsterdam, The Netherlands) as stimulus. As controls CD4^+^ T cells or adipocytes alone were cultured in presence or absence of stimulus. After overnight culture Brefeldin A (10 μg/mL) was added for 5 hours after which CD4^+^ T cells were harvested and intracellular staining for IL‐6 was performed.

### Detection of cytokines

Cytokines were measured in supernatant of CD4^+^ T cells isolated from SVF and in supernatant of unstimulated and αCD3/αCD28 stimulated IL‐6^+^ and IL‐6^−^ T cells isolated from SVF using the Milliplex Human Cytokine / Chemokine kit (Millipore), the Bio‐Plex array reader and Bio‐Plex software, according to manufactures’ protocol.

### Statistical analysis

Wilcoxon's signed rank test was used to compare differences between groups. A *p* value ≤ 0.05 was considered statistically significant.

## Conflict of interest

The authors have declared no financial or commercial conflict of interest.

**Abbreviations**
HEC: highly expanded clones
IFP: infrapatellar fat pad
SCAT: subcutaneous adipose tissue
SVF: stromal vascular fraction
Th4: T helper cell

## Supporting information

Peer review correspondence

Supporting Information Figure 1
Supporting Information Figure 2
Supporting Information Figure 3
Supporting Information Figure 4
Supporting Information Figure 5
