# Supplementary material for: Functional and phenotypical analysis of IL‐6‐secreting CD4+ T cells in human adipose tissue
Source: Eur J Immunol. 2018 Jan 29;48(3):471–81. doi: 10.1002/eji.201747037 (PMC5873429; doi:10.1002/eji.201747037)
Supplement: Supplementary file 2 — Supporting Information Figure 1 Supporting Information Figure 2 Supporting Information Figure 3 Supporting Information Figure 4 Supporting Information Figure 5 [file EJI-48-471-s002.pdf]

European Journal of Immunology

Supporting Information for DOI 10.1002/eji.201747037

Anja J. de Jong, Sabrina Pollastro, Joanneke C. Kwekkeboom, Stefan N. Andersen, Annemarie L. Dorjée, Aleida M. Bakker, Fawaz Alzaid, Antoine Soprani, Rob G.H.H. Nelissen, Jan B. Mullers, Nicolas Venteclef, Niek de Vries, Margreet Kloppenburg, René E.M. Toes and Andreea Ioan-Facsinay

Functional and phenotypical analysis of IL-6-secreting $CD4^+$ T cells in human adipose tissue

© 2017 The Authors. *European Journal of Immunology* published by WILEY-VCH Verlag GmbH & Co. KGaA, Weinheim.

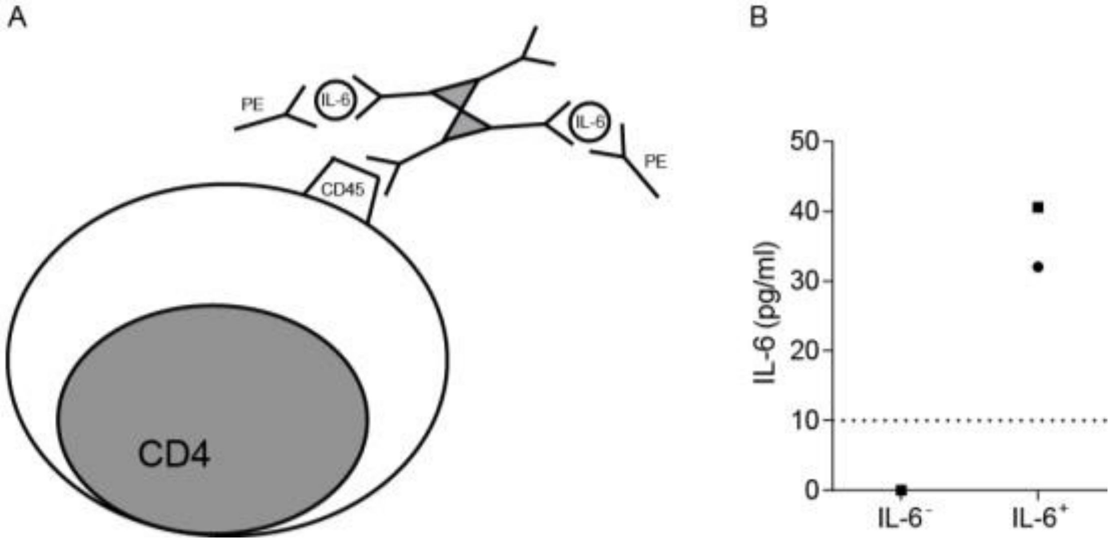

Supporting Figure 1. Capture complex

To isolate IL-6^{+} cells from IFP a capture complex was designed (A). Isolated IL-6^{+} CD4^{+} T cells (7744 or 966 cells/well) were able to produce IL-6 after isolation measured by ELISA (B).

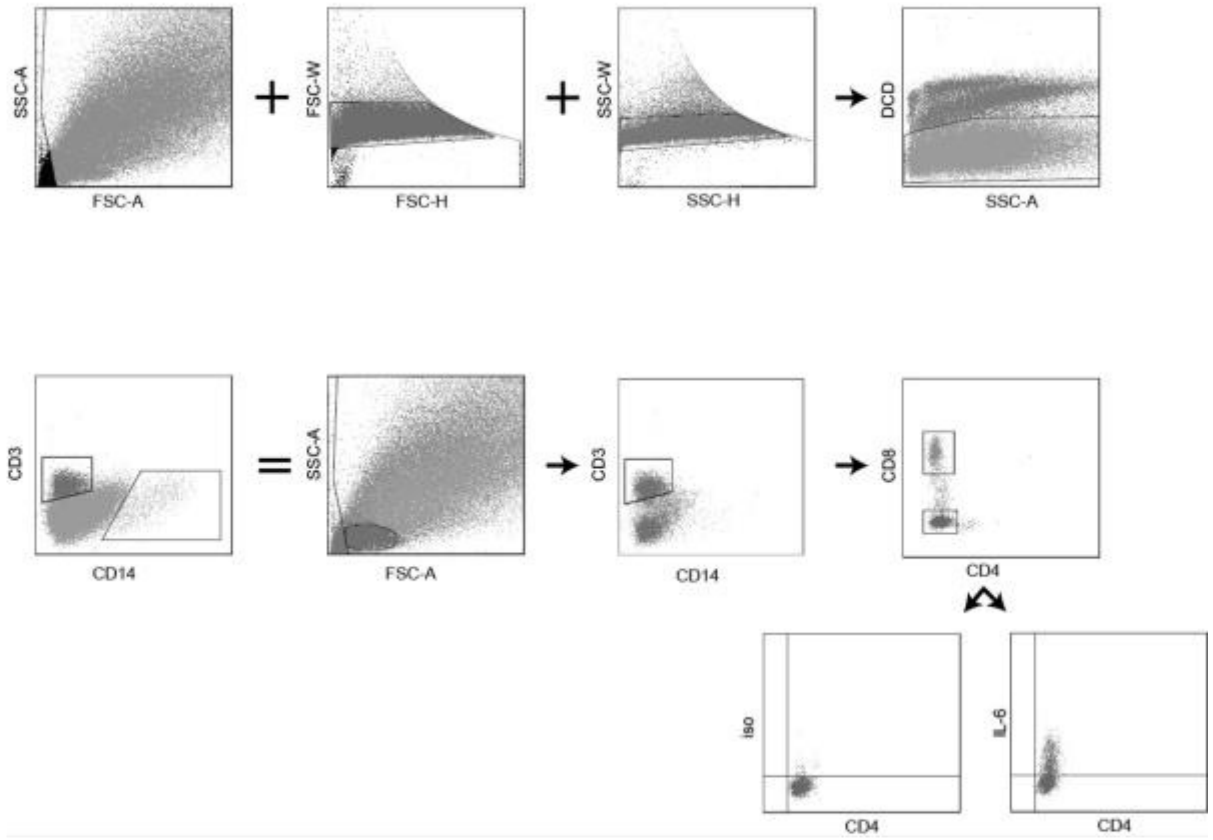

Supporting Figure 2. Gating strategy

A live gate based on FSC-A/SSC-A was set on SVF cells, where after two gates were set to exclude doublets. These gates were joined and dead cells were excluded. Using a CD3 positive gate lymphocytes were more strictly gated, followed by a CD4 positive gate to analyse IL-6 positive $CD4^+$ T cells.

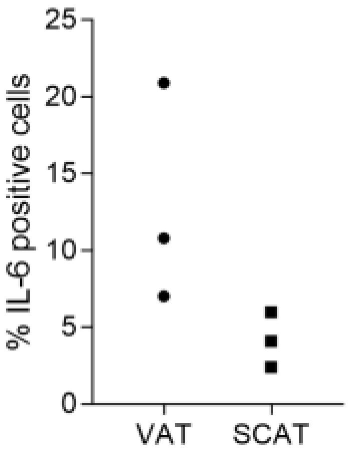

Supporting Figure 3. IL-6 positive T cells in visceral adipose tissue (VAT) and subcutaneous adipose tissue (SCAT)

The presence of IL-6$^+$ CD4$^+$ T cells was determined in visceral adipose tissue (VAT) and subcutaneous adipose tissue (SCAT) of patients undergoing bariatric surgery by flow cytometry (see gating strategy Supporting Information Fig 2) (N=3).

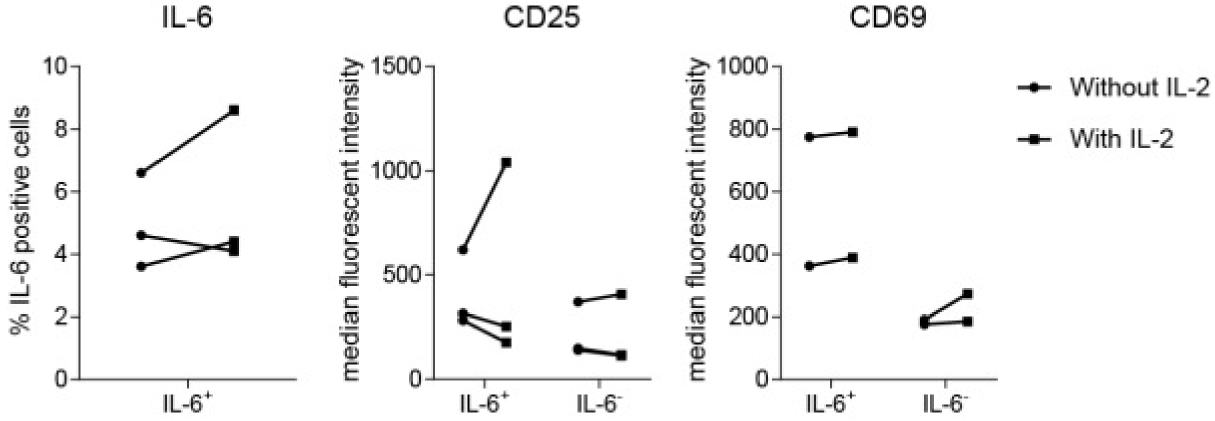

Supporting Figure 4. Expression levels of IL-6, CD25 and CD69 on SVF T cells upon overnight culture with or without IL-2

The percentage of IL-6^{+} CD4^{+} T cells in the presence and absence of IL-2 during the overnight culture was determined by flow cytometry (see gating strategy Supporting Information Fig. 2) (N=3). Furthermore, CD25 and CD69 expression on IL-6^{+} and IL-6^{-} CD4^{+} T cells was assessed (N=2-3).

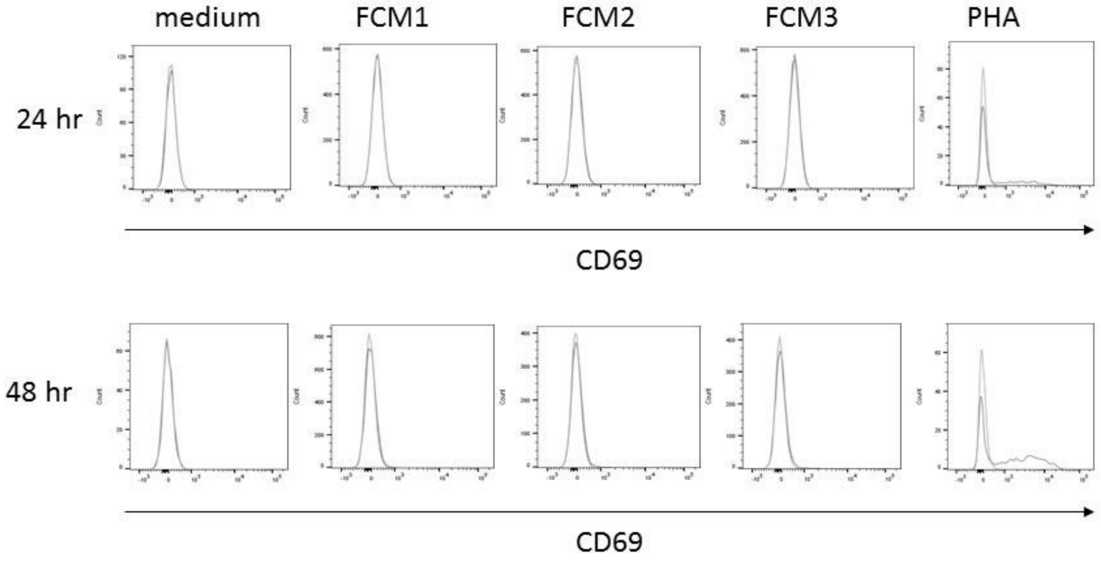

Supporting Figure 5. Induction of CD69 expression on CD4+ T cells by fat-conditioned medium from IFP (FCM)

Peripheral blood CD4+ T cells were cultured in the absence or presence of fat-conditioned medium (FCM) for 24 hrs or 48 hrs and CD69 expression levels were determined by flow cytometry (N=2, 6 different FCM were used). One donor is shown as example. Stimulation with PHA was used as positive control. Dark grey line represents isotype, light grey line represents CD69 staining.
