# Supplementary material for: Functional and phenotypical analysis of IL‐6‐secreting CD4+ T cells in human adipose tissue
Source: Eur J Immunol. 2018 Jan 29;48(3):471–81. doi: 10.1002/eji.201747037 (PMC5873429; doi:10.1002/eji.201747037)
Supplement: Supplementary file 1 — Peer review correspondence [file EJI-48-471-s001.pdf]

European Journal of Immunology

Functional and phenotypical analysis of IL-6-secreting CD4$^+$ T cells in human adipose tissue

Anja J. de Jong, Sabrina Pollastro, Joanneke C. Kwekkeboom, Stefan N. Andersen, Annemarie L. Dorjée, Aleida M. Bakker, Fawaz Alzaid, Antoine Soprani, Rob G.H.H. Nelissen, Jan B. Mullers, Nicolas Venteclef, Niek de Vries, Margreet Kloppenburg, René E.M. Toes, and Andreea Ioan-Facsinay
Correspondence: Dr. Andreea Ioan-Facsinay, Rheumatology, C1-R, Albinusdreef 2, 2333 ZA Leiden

---

Review Timeline:	Submission date:	10-Mar-2017
	First Editorial decision:	03-Apr-2017
	First revision received:	17-Oct-2017
	Second Editorial decision:	08-Nov-2017
	Second revision received:	27-Nov-2017
	Accepted:	22-Dec-2017

Handling Executive Committee member: Prof. Iain McInnes

Please note that the correspondence below does not include the standard editorial instructions regarding preparation and submission of revised manuscripts, only the scientific revisions requested and addressed.

First Editorial Decision
03-Apr-2017

Dear Dr. Ioan-Facsinay,

Manuscript ID eji.201747037 entitled "IL-6 secreting T cells in adipose tissue: a novel human CD4$^+$ T cell population" which you submitted to the European Journal of Immunology has been reviewed. The comments of the referees are included at the bottom of this letter.

You will see that both referees express concern about the credibility of the data, by virtue of either the method used/incomplete description of the methodology/approach, or the unusual results you report. You will see that one referee also feels that the novelty is limited. The Executive Editor therefore strongly encourages you to fully address the comments of both referees, for a revised version to be re-considered.

European Journal of Immunology

Should you disagree with any of the referees' concerns, you should address this in your point-by-point response and provide solid scientific reasons for why you will not make the requested changes.

You should also pay close attention to the editorial comments included below. *In particular, please edit your figure legends to follow Journal standards as outlined in the editorial comments. Failure to do this will result in delays in the re-review process.*

Please note that submitting a revision of your manuscript does not guarantee eventual acceptance, and that your revision will be re-reviewed by the referees before a decision is rendered.

If the revision of the paper is expected to take more than three months, please inform the editorial office. Revisions taking longer than six months may be assessed by new referee(s) to ensure the relevance and timeliness of the data.

Once again, thank you for submitting your manuscript to European Journal of Immunology and we look forward to receiving your revision.

Yours sincerely,
Karen Chu

On behalf of Prof. Iain McInnes

Dr. Karen Chu
Editorial Office
European Journal of Immunology
e-mail: ejied@wiley.com
www.eji-journal.eu

****

Reviewer: 1

Comments to the Author
The paper by de Jong et al. claims to have found a novel human IL-6 secreting T cell population that plays a role in adipose tissue. Although this concept, if supported by the data, would be new and of interest to the immunologic and rheumatologic scientific community, the paper suffers from several major flaws.

1) The actual cytokine production by the IFP T cells is extremely low. Cytokine levels (such as IL-6 and IL-8) that are secreted by tissue cells in synovial tissue are usually 10-100 fold higher, so the question is how relevant this observation is. In addition, in figure 1 no cell numbers are given so it is difficult to estimate what the relative contribution is.
2) In figure 1 and 2 the authors claim to have studied expression of spontaneously produced cytokines and activation markers. However, IL-2 was added to these cells. Thus the authors should proof the observed findings are not induced by IL-2. Both CD25 and CD69 can be induced by IL-2 and this presumably also holds true for IL-6 production
3) Percentages of IL-6 producing T cells are not higher in the tissue as compared to peripheral blood. This argues against an antigen-specific triggering of these T cells in the IFPs. In line with this no increased clonal expansion was found amongst IL6+ vs IL6- cells.
4) In view of the abovementioned remarks it is necessary that data are provided to demonstrate that specific inhibition/depletion of IL-6 producing T cells will somehow affect adipocytes functionally. In other words: demonstrate that these cells really make the difference
5) The authors suggest to have found a novel Th cell, secreting specifically IL-6 and state: “IL6+ Th cells cannot be categorized as a conventional Th subset”. However, the data that are provided are insufficient to demonstrate this. Eg. did the authors perform co-stainings with IL-17, IL-22, IL-9, IL-10 or even FoxP3. In particular since no functional activity of this specific subset in comparison with other subsets is shown the authors cannot make this statement. Hence, the title and entire manuscript should be adapted in this respect.
6) Histograms in Figure 3 are not convincingly showing chemokine receptor expression. It is unclear how, based upon the representative CCR4 expression, on average 40% of IL6+ T cells is CCR4 positive. (also description to the histograms are lacking)

Minor
In Figure 2 it would be of value to assess expression of the different markers on naïve, central memory, effector memory and effector Th cells (via CD45RO and CD27)

Reviewer: 2

Comments to the Author
This study investigates the production of IL-6 by T cells in the stromal vascular fraction of the infrapatellar fat pad in the knee of osteoarthritis patients. In addition to verifying the presence of IL-6+ T cell as already reported in a reference 17 (https://www.ncbi.nlm.nih.gov/pubmed/26980579) the authors shown that adipocytes can enhance IL-6 production by an unknown mechanism. The data are clear, and the methods appear to be technically sound. However, the results are hampered (a result of using the same clone for

purification and flow cytometry analysis?) and hard to interpret by the fact that the authors use no positive identification of CD4+ T cells, see below (from the gating strategy in sup fig. 2). Even though the IL-6 positive T cells are shown to be negative of HLA-DR (fig. 2), the authors have to address if the IL-6 producing cells are indeed CD4+ T cells or not “contaminating” cells as e.g. NKT cells, ILC cells, B cells or different kinds of CD14-APCs ?

The title indicates that the authors have identified a novel CD4+ T cell population. However, the authors need to show data on other hallmark cytokines as IL-17A (Th17) and IL-21 (Tfh) as well as master transcription factors to justify the title. Otherwise, should the title be moderated.

This manuscript requires some amendments before being in a publishable form.

Abstract:

-The authors conclude that the IL-6+ T cells display an activated phenotype of CD40L, CD69, CD25 and HLA-DR expression. In fig 2 they only show a significant (p=0.03) difference of CD69. The authors should tone down the conclusion.

-Have to be more specific in regards to the description of the expression of cytokines and chemokines in the IL-6+/- cells.

Introduction:

- Have to specify the Th subsets that are “pro-inflammatory” in sentence line 69-70.

Results:

- The authors state in line 104-105 “All IL-6+ CD4+ T cells expressed TCRαβ and
CD45RO (Fig. 2A) indicating that they are conventional memory T cells.” From fig. 2 it looks like the isotype (light grey ?) have an overlap with the specific staining of TCRαβ and CD45RO. The authors should moderate the conclusions.

- A comment on the CD69 tissue homing investigation is that CD69, in addition, to being expressed by tissue resident T cells is a well-recognized activation marker.

- Regarding the chemokine data in fig. 3, in the sentence line 121-124 the authors conclude “Furthermore, chemokine receptor expression showed that IL-6 producing T cells expressed a variety of chemokine receptors (Fig. 3B), precluding their unambiguous assignment to a certain T helper subset defined by their chemokine receptor expression. These data suggest that IL-6+ CD4+ T cells are not a conventional T helper subset”. I agree with the authors that there are no differences in the expression of the investigated chemokines between IL-6+/- T cells. However, the authors have to discuss the interesting heterogenic chemokine pattern observed in the T cells as CCR4 (Th2 associated) are expressed by 10-90% of the cells, CCR6 (Th17 associated) are expressed by 0-60% of the cells, CXCR3 (Th1 associated) are expressed by 10-70% of the cells and CXCR5 (Tfh associated) are expressed by 0-80%

of the cells in relation to the statement that IL-6+ CD4+ T cells are not a conventional T helper subset.

- In line 126-127 the authors state “The fact that IL-6+ CD4+ T cells from SVF display an activated state suggests that they have recently encountered antigen”, could the observed up-regulation of CD69 instead be mediated by tissue factors as IL-1beta, TGF-beta, CCL20 or other ?

- To better understand the mechanism of the shown induction of IL-6 in section “Adipocytes enhance IL-6 production in CD4+ T cells”, the authors should investigate the release of relevant cytokines as e.g. IL-1beta and TGF-beta from the supernatant (if available) of these cultures.

Discussion

- The conclusion in line 151-152 “Their TCRβ repertoire is distinct from IL-6- CD4+ T cells and they do not simultaneously secrete other cytokines” should be toned down since the authors do not investigate the expression of other relevant cytokines as IL-17A (Th17) and IL-21(Tfh).

- Could the speculated secretion of CCL20 from adipose tissue in line 173-174 be linked to the observed high expression of CCR6 on the T cells?

M&M

- Can the results using the purified CD4+ T cells (95%) be explained by contaminating none t cells?

- The author’s needs to address if the used restimulation (ionomycin+PMA) of the T cells cultures did have an effect in the detection of cell-surface molecules.

- It is unclear what ratio of T cells and adipocytes the authors are using; line 311-312 “Isolated CD4+ T cells were cultured overnight at a density of 200.000 cells/well in 96-well plates in 200 µl medium. CD4+ T cells were co-cultured with 20 µl of isolated adipocytes”

First revision – authors’ response

17-Oct-2017

We thank all reviewers for their useful remarks, as they have led to a significantly improvement of our manuscript. Please find below a point-by-point reply to the reviewers’ comments.

European Journal of Immunology

Reviewer: 1

Comments to the Author
The paper by de Jong et al. claims to have found a novel human IL-6 secreting T cell population that plays a role in adipose tissue. Although this concept, if supported by the data, would be new and of interest to the immunologic and rheumatologic scientific community, the paper suffers from several major flaws.

Reviewer comment: 1) The actual cytokine production by the IFP T cells is extremely low. Cytokine levels (such as IL-6 and IL-8) that are secreted by tissue cells in synovial tissue are usually 10-100 fold higher, so the question is how relevant this observation is. In addition, in figure 1 no cell numbers are given so it is difficult to estimate what the relative contribution is.
Author response and action: We agree with the reviewer that the levels of IL-6 secreted by T cells from IFP are rather low, but the number of IL-6$^+$ cells was also low. For the patients depicted in suppl. figure 1, 7744 cells and 966 cells were used, which results in an IL-6 production per cell of 0.0005-0.003 pg/cell. On a per cell basis this is comparable to synoviocytes as these produce approx. 0.005- 0.006 pg/cell (our own observations). These data indicate that the IL-6 secreted by the T cells could be a significant contributor to the inflammatory process in the OA joint. We have added these data to the manuscript (line 99) and we discussed it briefly in the 'discussion' (line 227-229).
We thank the reviewer for pointing out the omission in Fig.1, we have added the cell numbers to the figure legends (line 528)

Reviewer comment: 2) In figure 1 and 2 the authors claim to have studied expression of spontaneously produced cytokines and activation markers. However, IL-2 was added to these cells. Thus the authors should proof the observed findings are not induced by IL-2. Both CD25 and CD69 can be induced by IL-2 and this presumably also holds true for IL-6 production.
Author response and action: Indeed, IL-2 is capable of inducing the expression of CD25 and CD69. Therefore, we examined whether addition of IL-2 could influence the expression of these markers, as well as the secretion of IL-6. We assessed CD25, CD69 and IL-6 expression after overnight culture in the absence or presence of IL-2 in three patients (please see below). The results indicated that the addition of IL-2 to the cultures of SVF cells did not alter significantly the expression levels of CD25, CD69 and IL-6, supporting the initial conclusion that IL-6 is produced without additional stimulation by IFP T cells and that these cells express the activation markers CD25 and CD69. We have added these results to the manuscript (line 118-122 and Supporting Information Figure 4).

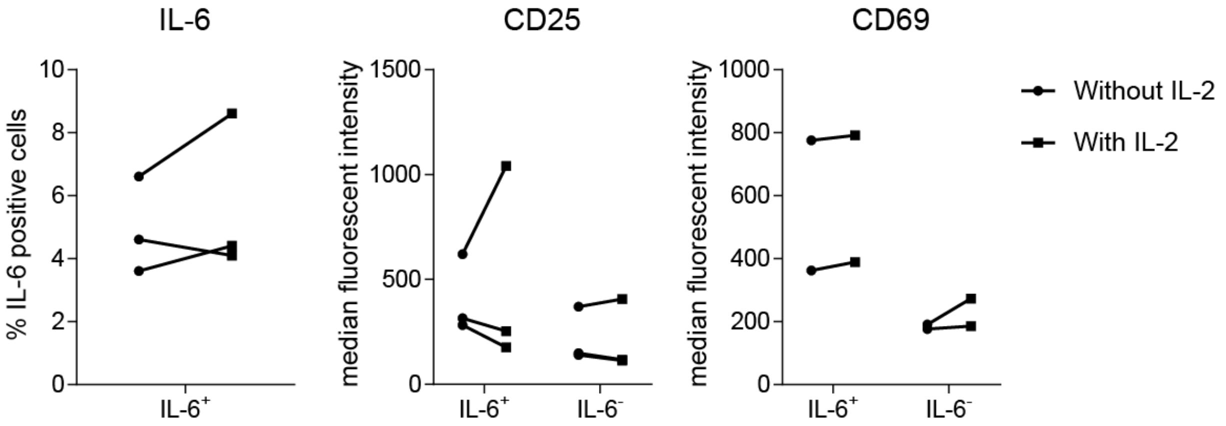

Reviewer comment: 3) Percentages of IL-6 producing T cells are not higher in the tissue as compared to peripheral blood. This argues against an antigen-specific triggering of these T cells in the IFPs. In line with this no increased clonal expansion was found amongst IL6+ vs IL6- cells.
Author response and action: We apologize for the confusion. Because we have included all samples in the overview figure (now Fig. 1E), it is somewhat unclear that the percentage IL-6 positive cells is higher in

tissue compared to blood. To make this clearer, we have added a figure (Fig. 1D) where we only show the IFP-blood paired samples. We hope that this figure conveys the message better.
We agree with the reviewer that there is no difference in clonal expansion between IL-6$^+$ and IL-6$^-$ T cells, indicating that both populations contain clonally-expanded cells. This however, does not exclude the possibility that IL-6$^+$ T cells have recently seen antigen. The fact that the majority of highly expanded clones are not shared between IL-6$^+$ and IL-6$^-$ T cells, this suggests that these cells recognize different epitopes and were primed under different conditions. We have changed the discussion accordingly (line 196-198).

Reviewer comment: 4) In view of the abovementioned remarks it is necessary that data are provided to demonstrate that specific inhibition/depletion of IL-6 producing T cells will somehow affect adipocytes functionally. In other words: demonstrate that these cells really make the difference
Author response and action: Although it would be very interesting to address this question, it is practically very challenging, as adipocytes survive only one day in culture and depletion of the IL-6 T cells would already take one day. Moreover, in our opinion, such experiments would warrant a separate study on its own. We are aware that this is a limitation of our study, therefore we have discussed their possible in vivo function in the 'discussion' section (line 227-229), mentioning also that this remains to be investigated in future studies.

Reviewer comment: 5) The authors suggest to have found a novel Th cell, secreting specifically IL-6 and state: "IL6+ Th cells cannot be categorized as a conventional Th subset". However, the data that are provided are insufficient to demonstrate this. Eg. did the authors perform co-stainings with IL-17, IL-22, IL-9, IL-10 or even FoxP3. In particular since no functional activity of this specific subset in comparison with other subsets is shown the authors cannot make this statement. Hence, the title and entire manuscript should be adapted in this respect.
Author response and action: We agree with the reviewer that additional data should be included to strengthen this statement. Therefore, we have assessed the secretion of several cytokines by isolated IL-6$^+$ T cells (Fig. 3B). These studies revealed that IL-6$^+$ T cells secrete only IL-6 in the absence of extra stimulation. Upon stimulation with anti-CD3/CD28, these cells are capable of secreting TNFα and very low amounts of IL-10 in addition to IL-6, but no IFNγ, IL-17A, IL-22, IL-9, IL-21, IL-4, IL-5 (Fig. 3B). Furthermore, we performed transcription factor analysis on groups of 20 sorted cells from a patient. These studies revealed that most IL-6$^+$ T cells (4 out of 7 groups of 20 cells) did not express any of the tested transcription factors (FoxP3, T-bet, GATA3, RORγt, Bcl6), while 6/6 groups of 20 IL-6$^-$ T cells expressed at least one transcription factor. Since we did find that 1 IL-6$^+$ group expressed FoxP3, 1 expressed both Bcl6 and T-bet and 1 only T-bet, we cannot exclude that part of the IL-6 producing cells could be Treg, Th1 or Tfh cells. Nevertheless, the majority of the IL-6$^+$ T cells could not be assigned to any conventional Th subset. We have added these data to the manuscript (Fig. 3C and line 139-145 in Results, line 189-194 in Discussion).
We do realize, however, that these data are obtained with a limited number of patients. Therefore, we have discussed this limitation in the 'Discussion' section.

Reviewer comment: 6) Histograms in Figure 3 are not convincingly showing chemokine receptor expression. It is unclear how, based upon the representative CCR4 expression, on average 40% of IL6+ T cells is CCR4 positive. (also description to the histograms are lacking)
Author response and action: We apologize for the unfortunate choice of the representative histograms. The percentages of CCR4 positive cells is very variable in our samples (between 20-90%), as indicated in the summary figure. It is, therefore, difficult to choose a truly representative example. Moreover, the number of IL-6 positive cells is very variable, which also influences the quality of the histogram representations. We have now included another histogram, which shows a higher percentage of CCR4 positive IL-6$^+$ T cells. We have also modified the histogram for CCR6 to better reflect the IL-6$^+$ population.
A description of the histograms has now been added in the legends of Fig. 2 and 3.
Minor
Reviewer comment: In Figure 2 it would be of value to assess expression of the different markers on naïve, central memory, effector memory and effector Th cells (via CD45RO and CD27)
Author response and action: We agree that this would be very interesting to assess, However, as the percentage of IL-6 positive cells and the amount of material we obtain is very limited, it is technically

impossible to visualize enough cells in each population to allow for a comparison.

Reviewer: 2

Comments to the Author
Reviewer comment: This study investigates the production of IL-6 by T cells in the stromal vascular fraction of the infrapatellar fat pad in the knee of osteoarthritis patients. In addition to verifying the presence of IL-6+ T cell as already reported in a reference 17 (https://www.ncbi.nlm.nih.gov/pubmed/26980579) the authors shown that adipocytes can enhance IL-6 production by an unknown mechanism. The data are clear, and the methods appear to be technically sound. However, the results are hampered (a result of using the same clone for purification and flow cytometry analysis?) and hard to interpret by the fact that the authors use no positive identification of CD4+ T cells, see below (from the gating strategy in sup fig. 2). Even though the IL-6 positive T cells are shown to be negative of HLA-DR (fig. 2), the authors have to address if the IL-6 producing cells are indeed CD4+ T cells or not "contaminating" cells as e.g. NKT cells, ILC cells, B cells or different kinds of CD14-APCs ?
Author response and action: We thank the reviewer for this comment, as it made us realize that the gating strategy we presented in the manuscript was not correct. After exclusion of doublets and dead cells, CD3+ CD14- cells were selected and gated back in FSC/SSC. This new gate was then used to select again the CD3+ CD14- cells and, within this population, the CD4+ cells. The expression of CD4 is usually lower in tissue than in blood T cells, which is indeed what we also observed. Despite the fact that we are confident that the cells we selected are CD3+, we can never completely exclude the possibility of a small population of contaminating cells. However, the fact that the IL-6^{+} cells express TCRαβ and CD3 and can be induced to secrete TNFα with a T cell specific stimulus (Fig. 3B), further substantiate that they are T cells. Likewise, all cells are positive for CD3 and IL-6 by FACS, indicating that, even if there are some contaminating cells present in this population, most of the IL-6 secreting cells are T cells.

Reviewer comment: The title indicates that the authors have identified a novel CD4+ T cell population. However, the authors need to show data on other hallmark cytokines as IL-17A (Th17) and IL-21 (Tfh) as well as master transcription factors to justify the title. Otherwise, should the title be moderated.
Author response and action: We agree with the reviewer that additional data should be included to strengthen this statement. Therefore, we have assessed the secretion of several cytokines by isolated IL-6^{+} T cells (Fig. 3B). These studies revealed that IL-6^{+} T cells secrete only IL-6 in the absence of extra stimulation. Upon stimulation with anti-CD3/CD28, these cells are capable of secreting TNFa and very low amounts of IL-10 in addition to IL-6, but no IFNγ, IL-17A, IL-22, IL-9, IL-21, IL-4, IL-5 (Fig. 3B). Furthermore, we performed transcription factor analysis on groups of 20 sorted cells from a patient. These studies revealed that most IL-6^{+} T cells (4 out of 7 groups of 20 cells) did not express any of the tested transcription factors (FoxP3, T-bet, GATA3, RORγt, Bcl6), while 6/6 groups of 20 IL6^{-} cells expressed at least one transcription factor. Since we did find that 1 IL-6^{+} group expressed FoxP3, 1 expressed both Bcl6 and T-bet and 1 only T-bet, we cannot exclude that part of the IL-6 producing cells could be Treg, Th1 or Tfh cells. Nevertheless, the majority of the IL-6^{+} T cells could not be assigned to any conventional Th subset. We have added these data to the manuscript (Fig. 3C and line 139-145 in Results, line 189-194 in Discussion).
We do realize, however, that these data are obtained with a limited number of patients. Therefore, we have discussed this limitation in the 'Discussion' section.

This manuscript requires some amendments before being in a publishable form.
Abstract:
Reviewer comment: -The authors conclude that the IL-6+ T cells display an activated phenotype of CD40L, CD69, CD25 and HLA-DR expression. In fig 2 they only show a significant (p=0.03) difference of CD69. The authors should tone down the conclusion.
Author response and action: We agree with the reviewer and have reformulated the conclusion.

Reviewer comment: -Have to be more specific in regards to the description of the expression of cytokines and chemokines in the IL-6+/- cells.

Author response and action: As the word limit does not allow the inclusion of extensive details on all markers tested, we have now specified that we have investigated cytokines, chemokine receptors and transcription factors associated with different conventional Th subsets and we specified these subsets.
Introduction:
Reviewer comment: - Have to specify the Th subsets that are “pro-inflammatory” in sentence line 69-70.
Author response and action: We have now included this information in the ‘introduction’ (line 75)

Results:
Reviewer comment: - The authors state in line 104-105 “All IL-6+ CD4+ T cells expressed TCRαβ and CD45RO (Fig. 2A) indicating that they are conventional memory T cells.” From fig. 2 it looks like the isotype (light grey ?) have an overlap with the specific staining of TCRαβ and CD45RO. The authors should moderate the conclusions.
Author response and action: We agree that a part of the specific staining overlaps with the isotype. However, as the whole population of T cells shifts in the actual staining compared to isotype, this indicates that all IL-6$^+$ CD4$^+$ T cells express TCRαβ and CD45RO. This is different than the expression of CD28 or CD25, in which two different populations can be observed, allowing for identification of a negative and a positive population of cells.

Reviewer comment: - A comment on the CD69 tissue homing investigation is that CD69, in addition, to being expressed by tissue resident T cells is a well-recognized activation marker.
Author response and action: We agree with the reviewer. We have now made this clearer in the revised manuscript (line 128).

Reviewer comment: - Regarding the chemokine data in fig. 3, in the sentence line 121-124 the authors conclude “Furthermore, chemokine receptor expression showed that IL-6 producing T cells expressed a variety of chemokine receptors (Fig. 3B), precluding their unambiguous assignment to a certain T helper subset defined by their chemokine receptor expression. These data suggest that IL-6+ CD4+ T cells are not a conventional T helper subset”. I agree with the authors that there are no differences in the expression of the investigated chemokines between IL-6+/- T cells. However, the authors have to discuss the interesting heterogenic chemokine pattern observed in the T cells as CCR4 (Th2 associated) are expressed by 10-90% of the cells, CCR6 (Th17 associated) are expressed by 0-60% of the cells, CXCR3 (Th1 associated) are expressed by 10-70% of the cells and CXCR5 (Tfh associated) are expressed by 0-80% of the cells in relation to the statement that IL-6+ CD4+ T cells are not a conventional T helper subset.
Author response and action: We agree with the reviewer that both IL-6$^+$ and IL-6$^-$ T cells from IFP display a variety of chemokine receptors. This could indicate that IL-6$^+$ T cells (and IL-6$^-$ T cells) are a mixture of several T helper subsets. However, based on the transcription factor and cytokine analyses, this seems unlikely, at least for the IL-6$^+$ T cells population. We have addressed this possibility in the discussion of the revised manuscript (line 185-194).

Reviewer comment: - In line 126-127 the authors state “The fact that IL-6+ CD4+ T cells from SVF display an activated state suggests that they have recently encountered antigen”, could the observed up-regulation of CD69 instead be mediated by tissue factors as IL-1beta, TGF-beta, CCL20 or other ?
Author response and action: We thank the reviewer for this suggestion. To address this possibility, we have studied whether soluble factors secreted by IFP could induce upregulation of CD69 in peripheral blood CD4 T cells. By using 2 different CD4$^+$ T cells donors each cultured with 3 different fat-conditioned media (FCM generated by culturing IFP explants for 24h), we concluded that CD69 expression was unaffected by adipose tissue-secreted factors, supporting the hypothesis that CD69 expression on IL-6$^+$ T cells could indeed reflect antigen encounter. We have included these data in our manuscript (Supplementary Fig. 5 and line 123-128)

Reviewer comment: - To better understand the mechanism of the shown induction of IL-6 in section “Adipocytes enhance IL-6 production in CD4+ T cells”, the authors should investigate the release of relevant cytokines as e.g. IL-1beta and TGF-beta from the supernatant (if available) of these cultures.
Author response and action: Unfortunately, we don’t have supernatant available of these cultures and could not perform analysis of IL-1beta and TGF-beta. We did, however, indicate the necessity to

investigate the adipocyte-derived signals that enhance IL-6 production in T cells in our discussion (line 216-217).

Discussion

Reviewer comment: - The conclusion in line 151-152 "Their TCRβ repertoire is distinct from IL-6- CD4+ T cells and they do not simultaneously secrete other cytokines" should be toned down since the authors do not investigate the expression of other relevant cytokines as IL-17A (Th17) and IL-21(Tfh).
Author response and action: We agree with the reviewer that additional cytokines should be investigated. Therefore, we have now performed milliplex analysis of IL-6$^+$ T cells and observed that they indeed do not secrete other cytokines associated with conventional Th cell subsets (Fig.3B), except for TNFα, which can be secreted by several Th subtypes. Moreover, we have performed transcription factor analyses, which indicate that the majority of the IL-6$^+$ T cells do not express any of the transcription factors associated with conventional Th subsets. This information has been added to the manuscript (Fig. 3B, 3C and line 139-147).

Reviewer comment: - Could the speculated secretion of CCL20 from adipose tissue in line 173-174 be linked to the observed high expression of CCR6 on the T cells?
Author response and action: Although this would be a possible mechanism, we did not investigate this association. However, we discuss this possible association in the revised manuscript (line 204-206).

M&M
Reviewer comment: - Can the results using the purified CD4+ T cells (95%) be explained by contaminating none t cells?
Author response and action: We assume that the reviewer refers to the experiments depicted in Fig. 5. We cannot fully exclude that contaminating cells could contribute to the observed results as populations of cells are never 100% pure after isolation. However, we consider it highly unlikely that contaminating cells explain the results because we first selected the lymphocytes based on FSC/SSC (resulting in exclusion of a small population of contaminating cells with a higher FSC and SSC), then we excluded doublets and dead cells and then we identified the IL-6-producing cells.

Reviewer comment: - The author's needs to address if the used restimulation (ionomycin+PMA) of the T cells cultures did have an effect in the detection of cell-surface molecules.
Author response and action: Cell surface molecules were assessed in the absence of stimulation. We have made this clearer in the revised manuscript (legend Fig. 2 and 3).

Reviewer comment: - It is unclear what ratio of T cells and adipocytes the authors are using; line 311-312 "Isolated CD4+ T cells were cultured overnight at a density of 200.000 cells/well in 96-well plates in 200 µl medium. CD4+ T cells were co-cultured with 20 µl of isolated adipocytes"
Author response and action: Based on the average volume of the adipocytes, there are approximately 75.000 adipocytes in 20 ul. The volume of the adipocytes was determined by light microscopy using an ocular ruler with which the diameter can be measured and the volume can be calculated by assuming that the adipocytes are spheres. 100 adipocytes were measured per donor and an average volume was calculated.

Second Editorial Decision

08-Nov-2017

08-Nov-2017

Dear Dr. Ioan-Facsinay,

Thank you for submitting your revised manuscript ID eji.201747037.R1 entitled "IL-6 secreting T cells in adipose tissue: a novel human CD4$^+$ T cell population" to the European Journal of Immunology. Your manuscript has been re-reviewed and the comments of the referees are included at the bottom of this letter.

Although the referees have recommended publication, some revisions to your manuscript have been requested.
Referee #1 expressed some concerns about the claim “to have found a novel IL-6-secreting Th subset”. For such reason the referee requested either additional experiments or, in alternative, to tone down the claim. Our Executive Editor is in agreement with these requests.

Therefore, I invite you to respond to the comments of the referees and revise your manuscript accordingly.

You should also pay close attention to the editorial comments included below. **In particular, please edit your figure legends to follow Journal standards as outlined in the editorial comments. Failure to do this will result in delays in the re-review process.**

If the revision of the paper is expected to take more than three months, please inform the editorial office. Revisions taking longer than six months may be assessed by new referees to ensure the relevance and timeliness of the data.

Once again, thank you for submitting your manuscript to European Journal of Immunology. We look forward to receiving your revision.

Yours sincerely,
Marta Vuerich

on behalf of
Prof. Iain McInnes

Editorial Office
European Journal of Immunology
e-mail: ejied@wiley.com

****

Reviewer: 2

Comments to the Author
No further comments to the manuscript.

Reviewer: 1

Comments to the Author
The provided extra data and the performed experiments by de Jong et al. improved the manuscript. There is only one concern that relates to the claim of the authors to have found a novel IL-6-secreting Th subset. Although the authors have provided some data to suggest that IL-6-secreting Th cells represent a unique population the data are not convincing enough.
Figure 3A only shows lack of co-expression of IL-6 with IFNg, TNFa and IL-4. Similar analyses should also be performed for IL-9, IL-17, IL-22, IL-10 (TGFb) and CXCR5. Such analyses have to be performed since the low number of cells hampers conclusions drawn from measurements of sorted cells. This "skews" the data since the most abundant cytokines (and transcription factors) will be detected, but not the less abundant ones.
Alternatively, if cell numbers are a drawback here the evidence for a unique IL-6-producing T cell subset could also come from sorting of the circulating counterparts. This would allow comparison of robust numbers of Th cells and their secreted cytokines.
A third option would be to remove the claim from the manuscript including the title. (eg. "Functional and phenotypical analysis of IL-6-secreting Th cells from adipose tissue")

Second revision – authors' response
27-Nov-2017

We want to thank the reviewers again for reviewing our manuscript. Please find below a reply to both reviewers.

Reviewer: 2

Comments to the Author
Reviewer comment: No further comments to the manuscript.

Author response and action: Thank you for your input.

Reviewer: 1

Comments to the Author
Reviewer comment: The provided extra data and the performed experiments by de Jong et al. improved the manuscript. There is only one concern that relates to the claim of the authors to have found a novel IL-6-secreting Th subset.
Although the authors have provided some data to suggest that IL-6-secreting Th cells represent a unique population the data are not convincing enough.
Figure 3A only shows lack of co-expression of IL-6 with IFNg, TNFa and IL-4. Similar analyses should also be performed for IL-9, IL-17, IL-22, IL-10 (TGFb) and CXCR5. Such analyses have to be performed since the low number of cells hampers conclusions drawn from measurements of sorted cells. This "skews" the data since the most abundant cytokines (and transcription factors) will be detected, but not the less abundant ones.
Alternatively, if cell numbers are a drawback here the evidence for a unique IL-6-producing T cell subset could also come from sorting of the circulating counterparts. This would allow comparison of robust numbers of Th cells and their secreted cytokines.
A third option would be to remove the claim from the manuscript including the title. (eg. "Functional and phenotypical analysis of IL-6-secreting Th cells from adipose tissue").

Author response and action: Thank you for your response. We agree with the reviewer and have toned down our conclusion and adjusted the title of the manuscript.

Third Editorial Decision

14-Dec-2017

Dear Dr. Ioan-Facsinay,

It is a pleasure to provisionally accept your manuscript entitled "Functional and phenotypical analysis of IL-6-secreting $CD4^+$ T cells in human adipose tissue" for publication in the European Journal of Immunology. For final acceptance, please follow the instructions below and return the requested items as soon as possible as we cannot process your manuscript further until all items listed below are dealt with.

Please note that EJI articles are now published online a few days after final acceptance (see Accepted Articles: http://onlinelibrary.wiley.com/journal/10.1002/(ISSN)1521-4141/accepted). The files used for the Accepted Articles are the final files and information supplied by you in Manuscript Central. You should therefore check that all the information (including author names) is correct as changes will NOT be permitted until the proofs stage.

We look forward to hearing from you and thank you for submitting your manuscript to the European Journal of Immunology.

European Journal of Immunology

Yours sincerely,

Eloho Etemire

on behalf of

Prof. Iain McInnes

Dr. Eloho Etemire
Editorial Office
European Journal of Immunology
e-mail: ejied@wiley.com
www.eji-journal.eu
